# Blood–Brain Barrier Dysfunction, Edema Formation and Functional Recovery in Ischemic and Hemorrhagic Stroke: A Retrospective Study

**DOI:** 10.3390/neurolint17110177

**Published:** 2025-11-01

**Authors:** Christian A. Müller, Jochen A. Sembill, Bernd Kallmünzer, Maximilian Bailer, Ludwig Singer, Tobias Engelhorn, Arnd Dörfler, Stefan Schwab, Stefanie Balk, Maximilian I. Sprügel

**Affiliations:** 1 Department of Neurology, Friedrich-Alexander-Universität Erlangen-Nürnberg (FAU), 91054 Erlangen, Germany; 2 Department of Neuroradiology, Friedrich-Alexander-Universität Erlangen-Nürnberg (FAU), 91054 Erlangen, Germany

**Keywords:** stroke, intracerebral hemorrhage, blood–brain barrier, cerebral edema, function recovery

## Abstract

Objectives: We aimed to determine temporal patterns of blood–brain barrier (BBB) dysfunction, edema formation and functional recovery in acute stroke. Materials and Methods: Patients of two observational studies on ischemic and hemorrhagic stroke between 2006 and 2019 were analyzed. Blood–brain barrier dysfunction was assessed using the cerebrospinal fluid-to-plasma albumin ratio. Edema formation was measured on all available imaging scans during hospital stay. Relative edema was defined as the ratio of edema volume to stroke volume. Multivariable regression models were applied to analyze associations and calculate predicted probabilities. Results: Overall, 138 stroke patients, 103 (74.6%) with ischemic stroke and 35 (25.4%) with hemorrhagic stroke, were analyzed. The predicted probability of substantial BBB dysfunction was approximately 46 (37–55) % among patients analyzed on 1 day after symptom onset and declined with increasing time, thereafter reaching 10 (3–29) % on day 30. The maximal extent of edema was lower in ischemic versus hemorrhagic stroke (relative edema: 1.5 [1.2–1.8] vs. 2.6 [1.9–4.5], *p* = 0.003) and occurred earlier after stroke onset (5.9 [4.6–8.5] days vs. 12.3 [9.7–16.4] days, *p* = 0.009). BBB dysfunction was associated with increased edema formation (adjusted relative edema: 4.0 [2.8–4.5] vs. 2.3 [1.8–3.0], *p* = 0.037) and lower chances of functional recovery (20/48 [41.7%] vs. 51/90 [56.7%], adjusted Odds Ratio: 0.37 [0.16–0.88], *p* = 0.025) in both ischemic and hemorrhagic stroke patients. Conclusions: BBB dysfunction frequently occurred in acute ischemic and hemorrhagic stroke and was associated with secondary injury and worse clinical outcomes. Future studies should evaluate BBB dysfunction as a potential therapeutic target using advanced imaging techniques early after stroke onset. Edema formation was aggravated and prolonged in hemorrhagic versus ischemic stroke.

## 1. Introduction

The blood–brain barrier (BBB) regulates leucocyte trafficking into the central nervous system (CNS), prevents invasion of pathogens and ion dysregulation and is essential to maintain neuronal function [1,2]. In experimental settings, stroke induces oxidative stress and disruption of tight junctions leading to BBB breakdown and aggravated stroke severity in animals [1,3,4,5]. However, it is rather unclear how frequently and at which time points substantial BBB dysfunction occurs after stroke onset in humans and whether it is associated with secondary injury and worse functional outcomes [1,2,5]. Furthermore, differences between ischemic and hemorrhagic strokes have been theorized, but even edema formation, representing a morphological imaging parameter for secondary stroke injury, has not been formally compared between ischemic and hemorrhagic stroke patients [6,7,8,9].

Advanced imaging techniques such as dynamic contrast enhanced magnetic resonance imaging provide valid assessment of BBB function in humans but are not performed in routine clinical practice [10]. BBB function may also be assessed in stroke patients using the cerebrospinal fluid (CSF)-to-plasma albumin ratio, as albumin represents a serum protein of large molecular weight which is prevented by the BBB from entering the brain under physiological conditions [11,12]. Albumin ratio has been evaluated in various diseases, providing insights into pathophysiological mechanisms of delirium and neurodegenerative diseases [1,2,3,11,13]. BBB dysfunction was previously reported by Prüss et al. in 33.1% of ischemic stroke patients, but has not been thoroughly investigated regarding temporal patterns, associations with secondary injury, and clinical outcomes and has been neglected in hemorrhagic stroke [13].

This study investigated (i) temporal patterns of substantial BBB dysfunction, (ii) temporal evolution of edema formation and (iii) the association between BBB dysfunction, edema and functional recovery in acute stroke, addressing potential differences between ischemic and hemorrhagic stroke patients.

## 2. Material and Methods

### 2.1. Study Participants

This retrospective analysis was performed following the Strengthening the Reporting of Observational Studies in Epidemiology (STROBE) reporting guidelines. Individual patient data of two longitudinal cohort studies were pooled comprising patients with ischemic stroke and patients with hemorrhagic stroke admitted to the Department of Neurology, University Hospital Erlangen, between 1 January 2006 and 31 December 2019. Patients with ischemic stroke receiving recanalization treatment (i.e., intravenous thrombolysis and/or endovascular therapy) and patients with large vessel occlusion (LVO) stroke not receiving recanalization treatment were included in the Universitätsklinikum Erlangen (UKER) ischemic stroke study [14,15,16]. Patients with hemorrhagic stroke (i.e., primary spontaneous intracerebral hemorrhage) were included in the UKER hemorrhagic stroke study [17,18,19]; patients with hemorrhagic stroke due to secondary etiology (e.g., trauma, aneurysm and tumor) were not included.

### 2.2. Clinical Variables and Definitions

Data on demographics, medical history, admission status, disease, treatment and imaging characteristics were obtained as previously published [14,15,16,17,18,19]. Ischemic stroke etiology was determined according to the Trial of Org 10172 in Acute Stroke Treatment (TOAST) classification and hemorrhagic stroke etiology was determined as anticoagulation, cerebral amyloid angiopathy (CAA), arterial hypertension or undetermined (for details see Supplemental Methods) [20,21]. Patients with intraventricular hemorrhage, CNS or other vasculitis, CNS or systemic infection, connective tissue disease, CNS neoplasia, brain surgery or traumatic LP were excluded due to potential implications to CSF parameters [22]. Intraventricular hemorrhage was defined as presence of any intraventricular blood on initial or follow-up imaging on day 2. Systemic infection was defined as white blood cell count >15,000/µL or C-reactive protein level >100 mg/L on the day that LP was performed, the day before or the day thereafter. Traumatic LP was defined as red blood cell count >5000/µL. Substantial blood–brain barrier dysfunction was defined as a cerebrospinal fluid-to-plasma albumin ratio above the age-adjusted upper reference limit calculated according to Hegen et al. (individual patient age divided by 25 plus 8) [23]. Functional recovery was defined as improvement of functional status by at least one point on the modified Rankin Scale between hospital discharge and 3 months after stroke onset [24].

### 2.3. Imaging Parameters

Stroke volume on day 2 was assessed on follow-up imaging routinely performed by non-contrast CT approximately 24 h after hospital admission using semi-automated software (syngo.via, Siemens, Erlangen, Germany, in ischemic stroke and Leonardo V, Siemens, Erlangen, Germany, in hemorrhagic stroke) [14,25,26]. In ischemic stroke patients without follow-up imaging on day 2 who did not receive recanalization treatment and did not show clinical improvement, stroke volume was assessed on initial imaging (perfusion lesion using the automated RAPID software, iSchemaView Inc., Menlo Park, Calif., USA; time to maximum of the residue function, Tmax > 6 s). In hemorrhagic stroke patients without follow-up imaging on day 2 who did not show clinical deterioration, stroke volume was assessed on initial imaging (volumetrically measured hematoma volume).

Edema in hemorrhagic stroke was measured on all available imaging scans during hospital stay as edema volume (mL) surrounding the bleeding using a semi-automatic volumetric algorithm [26]. Edema in ischemic stroke was measured on all available imaging scans during hospital stay as midline shift (mm) among those patients with reasonable likelihood of midline shift (stroke volume > 50 mL in the middle cerebral artery territory on day 2; only patients with midline shift >1 mm in at least one imaging scan after day 2 were included) [27,28] and converted into ml using the equation of the linear function between midline shift and lesion volume determined in a separate sample of 85 hemorrhagic stroke imaging scans (midline shift [mm] = 0.55 + 0.04 × lesion volume [mL]; Supplemental Figure S1). Relative edema was defined as the ratio of edema volume divided by stroke volume on day 2 [6]. Edema formation was analyzed for the subset of patients with at least 3 imaging scans performed during at least 3 different, previously established time points (1 day, 2 to 3 days, 4 to 6 days, 7 to 12 days, 13 to 18 days, 19 to 26 days and 27 to 35 days after symptom onset) [6].

### 2.4. Outcome Parameters

We assessed (i) temporal patterns of substantial BBB dysfunction, (ii) temporal evolution of edema formation and (iii) the association between BBB dysfunction, edema and functional recovery in ischemic and hemorrhagic stroke.

### 2.5. Statistical Analysis

SPSS version 28.0 (www.spss.com; IBM Corp., Armonk, NY, USA) and SAS/STAT version 15.3 (www.sas.com; SAS Institute, Cary, NC, USA) were used for statistical analyses. Categorial variables were compared using Pearson’s chi-square test and presented as counts (percentage). Continuous and ordinal variables were compared using the Mann–Whitney U-test and presented as median (interquartile range). Predicted probabilities of substantial BBB dysfunction were calculated using multivariable binary logistic regression analysis adjusted for continuous parameters identified in a univariate analysis to be associated (*p* < 0.10) with occurrence of substantial BBB dysfunction (age and stroke volume on day 2). Associations between substantial BBB dysfunction and functional recovery were determined by multivariable regression analysis adjusted for parameters associated with functional recovery (*p* < 0.10 in univariate analysis; age, stroke volume on day 2 and mRS at hospital discharge) and time from stroke onset to lumbar puncture. For edema analysis according to BBB dysfunction, edema values were adjusted for differences in edema formation between ischemic and hemorrhagic stroke using conversion factors calculated specifically for each of the 7 time points after stroke onset and for the maximal edema extent (for details, see Supplemental Methods).

## 3. Results

### 3.1. Study Population

**Figure 1:**
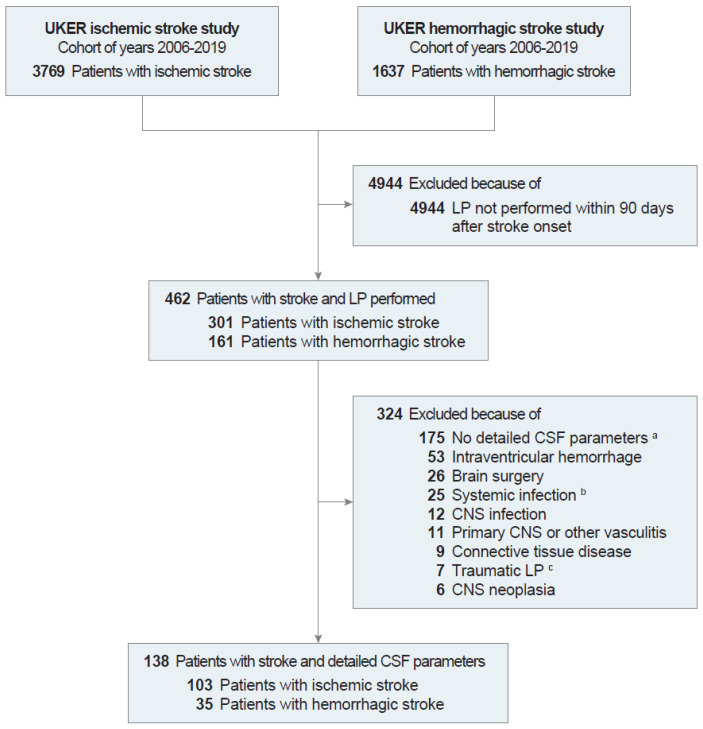
**Flow of study participants.** Patients without detailed CSF parameters and patients with characteristics potentially affecting CSF parameters (intraventricular hemorrhage, brain surgery, infection, vasculitis, connective tissue disease, traumatic LP and CNS neoplasia) were excluded [22]. ^a^ Detailed CSF parameters included albumin levels in CSF and blood. ^b^ Systemic infection was defined as white blood cell count >15,000/µL or C-reactive protein >100 mg/L on the day that LP was performed ±1 day. ^c^ Traumatic LP was defined as red blood cell count >5000/µL. Abbreviations: CNS, central nervous system; CSF, cerebrospinal fluid; LP, lumbar puncture; UKER, Universitätsklinikum Erlangen.

Among 5406 stroke patients, diagnostic LP was performed in 462 (8.5%) patients: 8.0% of ischemic stroke and 9.8% of hemorrhagic stroke patients (Figure 1). After exclusion of patients for disease and laboratory characteristics with potential effects on CSF parameters (e.g., intraventricular hemorrhage) and for incomplete CSF parameters (e.g., albumin values in CSF and blood), 138 stroke patients remained for final analysis: 103 (74.6%) ischemic stroke and 35 (25.4%) hemorrhagic stroke patients [22]. Patient characteristics are summarized in Table 1 and appear similar to established observational studies on ischemic and hemorrhagic stroke (Supplemental Tables S1 and S2). Maximal absolute edema was 61.4 [41.3–95.6] ml and maximal relative edema (i.e., maximal edema divided by stroke volume on day 2) was 2.0 [1.7–2.9] among 20 eligible stroke patients (see Methods Section). Substantial BBB dysfunction was present in 48 (34.8%) of the 138 stroke patients.

### 3.2. Temporal Patterns of Substantial BBB Dysfunction

**Figure 2:**
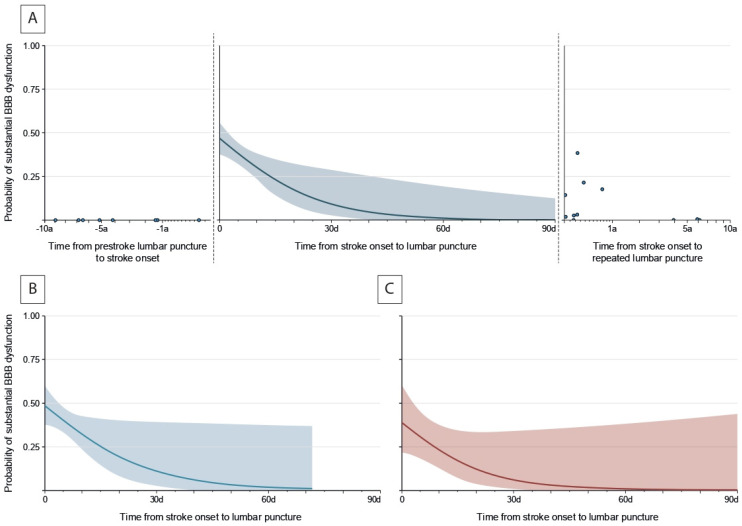
**Temporal patterns of substantial blood–brain barrier dysfunction.** (**A**) Predicted probability of substantial BBB dysfunction according to time from stroke onset to LP for (**left**) eight patients who had received LP years before stroke onset, (**middle**) 138 patients with acute stroke (presented as regression curve estimate for a typical patient aged 57 years with a stroke volume of 21.8 mL on day 2) and (**right**) eleven patients who had received repeated LPs after stroke. Predicted probabilities of substantial BBB dysfunction are presented for the subgroups of (**B**) 103 patients with acute ischemic stroke and (**C**) 35 patients with acute hemorrhagic stroke according to time from stroke onset to LP. Multivariable binary logistic regression analyses were adjusted for age and stroke volume on day 2.

The predicted probability of substantial BBB dysfunction was approximately 46 (37–55)% among patients analyzed one day after symptom onset (Figure 2B, adjusted for age and stroke volume on day 2) and declined with increasing time thereafter; 44 (36–52)% on day 2, 38 (32–45)% on day 5, 30 (22–38)% on day 10, 10 (3–29)% on day 30 and 0 (0–13)% on day 90 after symptom onset. Predicted probability of BBB dysfunction was 0% before stroke onset among the eight patients who had received separate lumbar puncture years before stroke onset. Predicted probability of BBB dysfunction declined and reached 0% several years after stroke onset among the eleven patients who had received repeated lumbar punctures after stroke. There were no significant differences between ischemic and hemorrhagic stroke patients regarding rate (38 of 103 [36.9%] in ischemic stroke vs. 10 of 35 [28.6%] in hemorrhagic stroke, *p* = 0.371) and temporal patterns (Figure 2B,C) of substantial BBB dysfunction. Stroke location and etiology were not significantly associated with the rate of substantial BBB dysfunction (*p* < 0.20).

### 3.3. Temporal Evolution of Edema Formation

**Figure 3:**
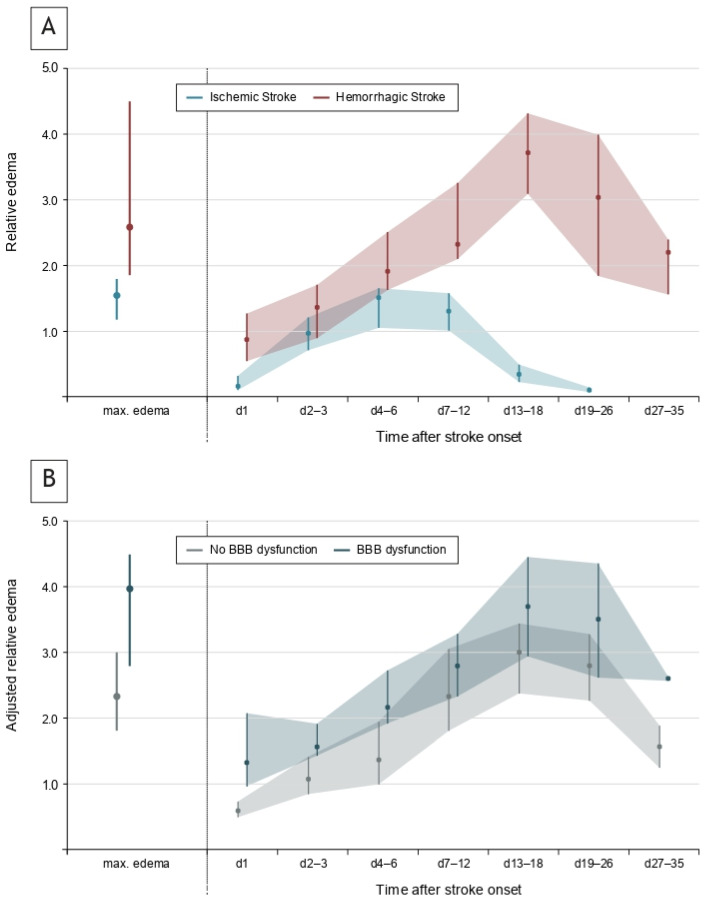
**Temporal evolution of edema formation.** (**A**) Relative edema values of patients with ischemic stroke (blue) and hemorrhagic stroke (red) for maximal edema extent (**left**) and for time points after stroke onset (**right**). (**B**) Relative edema values adjusted for stroke diagnosis (ischemic vs. hemorrhagic stroke) of patients without BBB dysfunction (gray) and patients with BBB dysfunction (blue).

Edema formation occurred gradually over days following stroke onset (Figure 3A). The median maximal extent was 61.4 (41.3–95.6) ml for absolute edema and 2.0 (1.7–2.9) for relative edema (i.e., ratio of edema volume and stroke volume) in 20 patients with serial edema measurement. The maximal extent of edema was lower in ischemic versus hemorrhagic stroke (1.5 [1.2–1.8] vs. 2.6 [1.9–4.5], *p* = 0.003) and occurred earlier after stroke onset (5.9 [4.6–8.5] days vs. 12.3 [9.7–16.4] days, *p* = 0.009).

### 3.4. Association Between BBB Dysfunction, Edema and Functional Recovery

Edema formation was significantly increased among patients with substantial BBB dysfunction (adjusted maximal edema: 4.0 [2.8–4.5] vs. 2.3 [1.8–3.0], *p* = 0.037) and edema formation associated with BBB dysfunction appeared early after stroke onset (Figure 3B; relative edema was adjusted for stroke diagnosis, i.e., ischemic versus hemorrhagic stroke). Rate of functional recovery was significantly lower among patients with substantial BBB dysfunction (20/48 [41.7%] vs. 51/90 [56.7%], adjusted Odds Ratio: 0.37 [0.16–0.88], adjusted absolute difference: −24.2 [−45.7 to −2.6] %, *p* = 0.025) after adjustment for age, stroke volume on day 2, mRS at hospital discharge and time from stroke onset to lumbar puncture. Stroke diagnosis had no significant interaction with the effect of BBB dysfunction on functional recovery (*p* = 0.945 for interaction).

## 4. Discussion

Substantial BBB dysfunction was present in approximately 46% of stroke patients within the first day after symptom onset and was associated with secondary injury and lower chances of functional recovery in this study. Edema formation was more pronounced and prolonged in hemorrhagic stroke patients, but frequency of BBB dysfunction and its impact on functional recovery were similar in ischemic and hemorrhagic stroke patients. Several aspects deserve attention as BBB dysfunction could represent a therapeutic target in future treatment strategies for both ischemic and hemorrhagic stroke patients.

Substantial BBB dysfunction occurred in approximately 46% of stroke patients immediately after stroke onset, but the incidence gradually declined within days thereafter, reaching 10% on day 30. This is an important consideration for future studies evaluating alternative detection methods and potential treatment strategies for BBB dysfunction. Regarding alternative detection methods, lumbar puncture is an interventional procedure associated with relatively low but potential risks, and BBB dysfunction should be evaluated using imaging techniques such as dynamic contrast enhanced MRI to avoid procedure-related adverse events [10]. However, our study results highlight that imaging scans must be performed within 24 h after stroke onset because incidence of BBB dysfunction would otherwise be falsely underestimated. Regarding potential treatment strategies, future interventions should be initiated within 24 h after stroke onset to achieve the highest possible treatment benefit. This aspect is further supported by the data on the temporal evolution of edema formation presented in this study suggesting that secondary injury associated with BBB dysfunction emerges early after stroke onset.

Edema formation was more pronounced in hemorrhagic stroke and peaked approximately 12 days after stroke onset compared to after 6 days among ischemic stroke patients in this study. Our results are in line with experience in clinical practice and previous studies investigating edema formation in ischemic or hemorrhagic stroke patients separately, but temporal evolution of edema formation has not been formally compared between ischemic and hemorrhagic stroke [6,7,8,9]. Measuring both edema volume and midline shift in large hemorrhagic stroke enabled us to convert midline shift into edema volumes in ischemic stroke and formally compare edema formation in ischemic versus hemorrhagic stroke. Future studies evaluating edema formation in stroke should consider the methodology and findings of our study.

Substantial BBB dysfunction was associated with lower chances of functional recovery after statistical adjustment for potential confounders in this study. The rate of functional recovery was 24% lower among patients with substantial BBB dysfunction suggesting a potential therapeutic target of high clinical significance. Future therapeutic agents attenuating BBB dysfunction might exert treatment effects comparable to thrombolysis in ischemic stroke. Although residual confounding should be considered, the association appears reasonable, corresponds to findings from experimental studies and is supported by data on edema formation, which was significantly increased among patients with substantial BBB dysfunction in this study [1,2,3,4,5]. Edema represents an imaging parameter of secondary injury in stroke, and the effect of BBB dysfunction on functional recovery of stroke patients appears to be mediated by secondary injury pathways [6,7,8]. As rate and temporal patterns of BBB dysfunction were similar in ischemic and hemorrhagic stroke patients, BBB dysfunction represents a promising treatment target for both hemorrhagic and ischemic stroke patients.

This study has several limitations. The retrospective and single-center study design and small patient numbers for edema analyses may lead to limited generalizability of our study’s findings. Lumbar puncture was performed only in approximately 9% of stroke patients, leading to potential confounding by indication, although patient characteristics in this study appear similar to established cohort studies in ischemic and hemorrhagic stroke. We statistically accounted for treatment interventions targeting functional recovery (e.g., endovascular therapy or thrombolysis for ischemic stroke, anticoagulation reversal or blood pressure reduction in hemorrhagic stroke) only by adjustment for stroke volume on day 2 which, however, mediates the effect on functional recovery [29,30,31,32]. Treatment interventions targeting edema formation (e.g., hypothermia and osmotherapy) were statistically not accounted for as they are infrequently applied and their treatment effects are questionable [33,34,35,36]. BBB dysfunction was determined solely based on cerebrospinal fluid-to-plasma albumin ratio, which appears to be a valid parameter of BBB function after stroke considering the values of LPs performed before stroke and values of repeated LPs performed after stroke, but future studies should evaluate imaging-based assessments of BBB dysfunction.

## 5. Conclusions

In summary, substantial BBB dysfunction occurred frequently in acute stroke and was associated with aggravated secondary injury and lower chances of functional recovery among both ischemic and hemorrhagic stroke patients. Future studies should further evaluate BBB dysfunction to predict secondary injury and functional recovery and explore a potential therapeutic target using advanced imaging techniques, notably on the first day after stroke onset.

## Figures and Tables

**Table 1:** Characteristics of study participants.

	Overall Stroke Patients (n = 138)
Age, years, median (IQR)	58 (46–73)
Female sex, n (%)	72 (52.2%)
Medical history, n (%)	
Atrial fibrillation	16 (11.6%)
Anticoagulation therapy	9 (6.5%)
Hypertension	88 (63.8%)
Diabetes mellitus	26 (18.8%)
Previous ischemic stroke or TIA	27 (19.6%)
Previous hemorrhagic stroke	5 (3.6%)
NIHSS score at hospital admission, median (IQR)	4 (2–9)
Treatment with intravenous alteplase, n (%)	38/103 (27.5%)
Treatment with endovascular therapy, n (%)	7/103 (5.1%)
Time from first observation of symptoms to hospital admission, hours, median (IQR)	8.6 (2.6–48.8)
Diagnosis, n (%)	
Ischemic stroke	103 (74.6%)
Hemorrhagic stroke	35 (25.4%)
Ischemic stroke subtype	
Cardioembolic disease	26/103 (25.2%)
Large-vessel disease	24/103 (23.3%)
Small-vessel disease	16/103 (15.5%)
Other cause ^a^	15/103 (14.6%)
Undetermined	22/103 (21.4%)
Hemorrhagic stroke subtype	
Hypertension	16/35 (45.7%)
Amyloid angiopathy	11/35 (31.4%)
Anticoagulation	5/35 (14.3%)
Undetermined	3/35 (8.6%)
Imaging characteristics	
Stroke volume on day 2, mL, median (IQR) ^b^	5.6 (2.5–21.6)
Stroke location	
Deep, n (%)	23 (16.7%)
Lobar, n (%)	102 (73.9%)
Infratentorial, n (%)	13 (9.4%)

^a^ Patients with primary CNS or other vasculitis, CNS infection, systemic infection and CNS neoplasia were excluded due to potential implications on CSF parameters [22]. ^b^ Stroke volume on day 2 was assessed on follow-up imaging performed at a median of 24.2 (19.1–28.6) hours after hospital admission using semi-automated software [14,25,26]. Abbreviations: TIA, transient ischemic attack; NIHSS, National Institutes of Health Stroke Scale.

## Data Availability

The datasets used and analyzed during the current study are available from the corresponding author upon request.

## Supplementary Materials

The following supporting information can be downloaded at https://www.mdpi.com/article/10.3390/neurolint17110177/s1: Supplemental Methods, Supplemental Figure S1, Supplemental Table S1, Supplemental Table S2. References [20,21,37,38,39,40,41,42] are cited in Supplementary Materials.
