# Supplementary material for: Blood–Brain Barrier Dysfunction, Edema Formation and Functional Recovery in Ischemic and Hemorrhagic Stroke: A Retrospective Study
Source: Neurol Int. 2025 Nov 1;17(11):177. doi: 10.3390/neurolint17110177 (PMC12655834; doi:10.3390/neurolint17110177)
Supplement: Supplementary file 1 [file neurolint-17-00177-s001.zip › neurolint-3917462-supplementary.pdf]

# SUPPLEMENTAL MATERIAL

Müller CA, Sembill JA, Kallmünzer B, et al. Blood–brain barrier dysfunction, edema formation and functional recovery in ischemic and hemorrhagic stroke: a retrospective study

**Supplemental Methods**

**Supplemental Figure S1:** **Association between midline shift and stroke lesion volume in hemorrhagic stroke**

**Supplemental Table S1:** **Clinical characteristics of ischemic stroke patients in the UKER ischemic stroke study and external observational studies**

**Supplemental Table S2:** **Clinical characteristics of hemorrhagic stroke patients in the UKER hemorrhagic stroke study and external observational studies**

This supplementary material has been provided by the authors to give readers additional information about their work.

## Supplemental Methods

Ischemic stroke etiology was determined according to the Trial of Org 10172 in Acute Stroke Treatment (TOAST) classification as large-vessel disease, cardioembolic, small-vessel disease, other determined cause and undetermined cause [1]. Hemorrhagic stroke etiology was determined as anticoagulation, cerebral amyloid angiopathy (CAA), arterial hypertension or undetermined [2]. Anticoagulation etiology was defined as treatment with vitamin K antagonist and international normalized value >1.5 on hospital admission or treatment with novel oral anticoagulants at hemorrhagic stroke onset [3]. CAA etiology was defined as definite or probable CAA according to the modified Boston or high risk CAA according to the simplified Edinburgh criteria in lobar hemorrhagic stroke [4, 5]. Arterial hypertension etiology was defined as elevated pre-stroke blood pressure, pre-stroke use of blood pressure medication or deep hemorrhagic stroke location in patients aged ≥40 years.

For edema analysis according to BBB dysfunction, edema values were adjusted for differences in edema formation between ischemic and hemorrhagic stroke (edema values of ischemic stroke patients were multiplied by the conversion factor calculated for each time point [i.e. median edema volume in hemorrhagic stroke divided by median edema volume in ischemic stroke]: day 1: 5.35; day 2-3: 1.41; day 4-6: 1.27; day 7-12: 1.78; day 13-18: 10.77; day 19-26: 29.21; maximal edema: 1.67).

**Supplemental Figure S1: Association between midline shift and stroke lesion volume in hemorrhagic stroke**

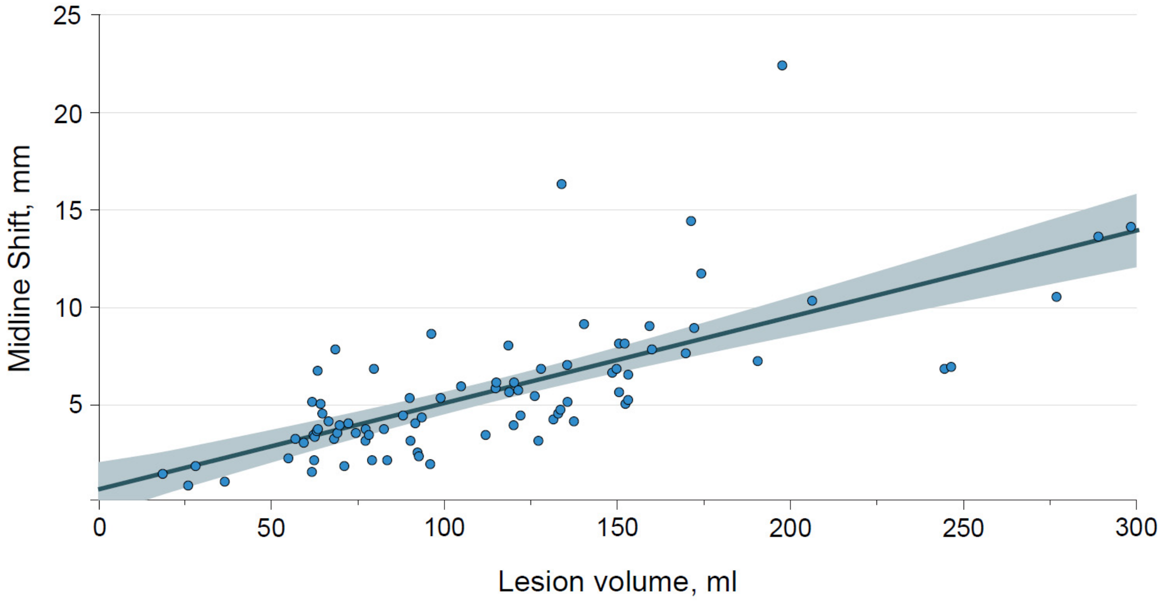


Linear association between midline Shift and stroke lesion volume (y=0.55+0.04*x). Midline Shift and stroke lesion volume (stroke volume plus edema volume) were measured on 85 imaging scans of 25 patients (between day 1 and day 31 after stroke onset) with hemorrhagic stroke and lesion volume between 18.6 and 297.6ml.

**Supplemental Table S1: Clinical characteristics of ischemic stroke patients in the UKER ischemic stroke study and external observational studies [6, 7]**

| | **UKER ischemic stroke study years 2006-2019 (n=103)** | **Northwestern Germany Stroke registry years 2016-2017 (n=146 062) [6]** | **GWTG-Stroke registry years 2016-2017 (n=348 715) [7]** |
|---|---|---|---|
| Age, years, mean (SD) | 56.6 (18.9) | 72.7 (13.1) | 70.1 (14.4) |
| Female Sex, n (%) | 54 (52.4%) | 69234 (47.4%) | 172338 (49.4%) |
| Medical history, n (%) | | | |
| Atrial fibrillation | 9 (8.7%) | 28962 (19.8%) | 71687 (20.6%) |
| Anticoagulation therapy | 3 (2.9%) | NA | 84848 (24.3%) |
| Hypertension | 60 (58.3%) | 124754 (85.4%) | 265847 (76.3%) |
| Diabetes Mellitus | 20 (19.4%) | 42944 (29.4%) | 122096 (35.0%) |
| Previous ischemic stroke or TIA | 23 (22.3%) | 38089 (26.1%) | 106524 (30.6%) |
| NIHSS score, median (IQR) | 4 (2-9) | 4 (2-6) | 4 (1-9) |
| Ischemic stroke subtype | | | |
| Cardioembolic disease | 26 (25.2%) | 46097 (31.6%) | 96592 (27.7%) |
| Large-vessel disease | 24 (23.3%) | 33314 (22.8%) | 81394 (23.3%) |
| Small-vessel disease | 16 (15.5%) | 30003 (20.5%) | 82542 (23.7%) |
| Other cause [a] | 15 (14.6%) | 5153 (3.5%) | 18330 (5.3%) |
| Undetermined | 22 (21.4%) | 31495 (21.5%) | 69857 (20.0%) |

[a] Other cause: 6 patients with dissection, 6 patients with coagulopathy, 3 patients with Moyamoya syndrome. Abbreviations: TIA, transient ischemic attack; NA, not available; NIHSS, National Institutes of Health Stroke Scale; UKER, Universitätsklinikum Erlangen.

**Supplemental Table S2: Clinical characteristics of hemorrhagic stroke patients in the UKER hemorrhagic stroke study and external observational studies [2, 8]**

| | **UKER hemorrhagic stroke study years 2006-2019 (n=35)** | **Ludwigshafen Stroke registry years 2006-2010 (n=152) [8]** | **Helsinki ICH study years 2005-2010 (n=1013) [2]** |
|---|---|---|---|
| Age, years, median (IQR) | 63 (52-73) | 74 (63-81) | 68 (58-78) |
| Female Sex, n (%) | 18 (51.4%) | 75 (49.3%) | 431 (42.5%) |
| Medical history, n (%) | | | |
| Atrial fibrillation | 7 (20.0%) | 13 (11.1%) | 142 (14.0%) |
| Anticoagulation therapy | 6 (17.1%) | 24 (15.8%) | 132 (13.0%) |
| Hypertension | 28 (80.0%) | 133 (90.5%) | 637 (62.9%) |
| Diabetes Mellitus | 6 (17.1%) | 34 (24.3%) | 143 (14.1%) |
| Previous ischemic stroke or TIA | 4 (11.4%) | NA | 146 (14.4%) |
| Previous hemorrhagic stroke | 4 (11.4%) | NA | 54 (5.3%) |
| NIHSS score at hospital admission, median (IQR) | 2 (1-6) | 10 (4-17) | 11 (4-20) |
| Hemorrhagic stroke subtype | | | |
| Hypertension | 16 (45.7%) | 73/138 (52.9%) | 354/915 (38.7%) |
| Amyloid angiopathy | 11 (31.4%) | 44/138 (31.9%) | 205/915 (22.4%) |
| Anticoagulation | 5 (14.3%) | 16/138 (11.6%) | 143/915 (15.6%) |
| Undetermined | 3 (8.6%) | 5/138 (3.6%) | 213/915 (23.3%) |

Abbreviations: ICH, intracerebral hemorrhage; TIA, transient ischemic attack; NA, not available; NIHSS, National Institutes of Health Stroke Scale; UKER, Universitätsklinikum Erlangen.

**References**

1. Adams HP, Jr., Bendixen BH, Kappelle LJ, et al. Classification of subtype of acute ischemic stroke. Definitions for use in a multicenter clinical trial. TOAST. Trial of Org 10172 in Acute Stroke Treatment. Stroke 1993;24:35-41.

2. Meretoja A, Strbian D, Putaala J, et al. SMASH-U: a proposal for etiologic classification of intracerebral hemorrhage. Stroke 2012;43:2592-2597.

3. Sprügel MI, Kuramatsu JB, Gerner ST, et al. Antiplatelet Therapy in Primary Spontaneous and Oral Anticoagulation-Associated Intracerebral Hemorrhage. Stroke 2018;49:2621-2629.

4. Charidimou A, Boulouis G, Frosch MP, et al. The Boston criteria version 2.0 for cerebral amyloid angiopathy: a multicentre, retrospective, MRI-neuropathology diagnostic accuracy study. The Lancet Neurology 2022;21:714-725.

5. Sembill JA, Knott M, Xu M, et al. Simplified Edinburgh CT Criteria for Identification of Lobar Intracerebral Hemorrhage Associated With Cerebral Amyloid Angiopathy. Neurology 2022;98:e1997-e2004.

6. Bonkhoff AK, Rübsamen N, Grefkes C, Rost NS, Berger K, Karch A. Development and Validation of Prediction Models for Severe Complications After Acute Ischemic Stroke: A Study Based on the Stroke Registry of Northwestern Germany. Journal of the American Heart Association 2022;11:e023175.

7. Prabhakaran S, Messé SR, Kleindorfer D, et al. Cryptogenic stroke: Contemporary trends, treatments, and outcomes in the United States. Neurology Clinical practice 2020;10:396-405.

8. Palm F, Henschke N, Wolf J, et al. Intracerebral haemorrhage in a population-based stroke registry (LuSSt): incidence, aetiology, functional outcome and mortality. Journal of neurology 2013;260:2541-2550.
